# 1191. Pediatric Long Haul Post COVID-19: Is New Jersey’s Experience Different?

**DOI:** 10.1093/ofid/ofab466.1383

**Published:** 2021-12-04

**Authors:** Aakriti Bhargava, Lauren Farrand, Stephen Zieniewicz, Dennis J Brenner, Timothy S Yeh, Uzma Hasan

**Affiliations:** 1 Saint Barnabas Medical Center, Carmel, Indiana; 2 Saint Barnabas Medical Center/Rutgers New Jersey Medical School, Livingston, New Jersey

## Abstract

**Background:**

COVID-19 impacted nearly 4 million children, accounting for 14% of total cases in the US, 1.3-3.2% of total reported hospitalizations and less than 1% deaths attributed to COVID-19. Many studies report persistent symptoms in adults several months after acute COVID-19. Similar findings have been reported from a small cohort of children in Italy. To date there are no studies reviewing long haul symptoms in children in the US.

**Methods:**

With the goal of defining long haul in pediatric population, and providing comprehensive care to these patients, RWJBarnabasHealth launched a post-COVID CARE program in October 2020 for children. The program has provided care for approximately 16 patients with COVID related Multisystem Inflammatory Syndrome (MIS-C) and 48 pediatric patients with COVID. The goal of the Pediatric Post-COVID CARE program was to provide a multidisciplinary approach for children ages 0-21 years impacted with COVID-19. This included patients who experienced ongoing symptoms >4 weeks from initial COVID-19 illness. All children were assessed by a pediatric infectious disease physician and triaged to appropriate subspecialties, all part of the long-haul care team. In addition, physical therapy and psychology support services were provided to facilitate return to normalcy.

**Results:**

To date, our program has evaluated 64 patients. 28% experienced at least 1 symptom 4 weeks after acute COVID-19. Median age was 14 years and 77.8% were female. The follow-up study was conducted from October 2020 to May 2021. Data was collected 2 weeks, 6 weeks, 3 months, and 6 months post discharge or initial evaluation in clinic. 28% of patients were antibody positive, 55.6% experienced fatigue, 50% experienced shortness of breath or cough, 50% experienced ‘brain fog’,33% chest pain and 44.4% experienced anxiety and/or depression.

**Conclusion:**

Early identification of patients and comprehensive protocols may facilitate return to normalcy for children with lingering somatic symptoms worsened by impact of social isolation, economic stresses, lost parental jobs, and food insecurity among many other contributing factors. Further research is needed to determine why children of certain ethnicities are impacted differently.

**Disclosures:**

**All Authors**: No reported disclosures

